# Determination of Critical Micellar Concentration of Homologous 2-Alkoxyphenylcarbamoyloxyethyl-Morpholinium Chlorides

**DOI:** 10.3390/molecules23051064

**Published:** 2018-05-02

**Authors:** Lenka Stopková, Jana Gališinová, Zuzana Šuchtová, Jozef Čižmárik, Fils Andriamainty

**Affiliations:** Department of Pharmaceutical Chemistry, Faculty of Pharmacy, Comenius University, Odbojárov 10, 83232 Bratislava, Slovakia; lenka.stopkova@gmail.com (L.S.); jana.galisinova@gmail.com (J.G.); zuzanasuchtova@gmail.com (Z.S.); cizmarik@fpharm.uniba.sk (J.C.)

**Keywords:** local anesthetic, morpholinium chloride, critical micellar concentration, pyrene absorption, sigmoidal Boltzmann equation

## Abstract

The critical micellar concentrations of selected alkyloxy homologues of local anesthetic 4-(2-{[(2-alkoxyphenyl)carbamoyl]oxy}ethyl)morpholin-4-ium chloride with *n_c_* = 2, 4, 5, 6, 7, 8, and 9 carbons in alkyloxy tail were determined by absorption spectroscopy in the UV–vis spectral region with the use of a pyrene probe. Within the homologous series of the studied amphiphilic compounds, the ln(*cmc*) was observed to be dependent linearly on the number of carbon atoms *n_c_* in the hydrophobic tail: ln(*cmc*) = 0.705–0.966 *n_c_*. The Gibbs free energy, necessary for the transfer of the methylene group of the alkoxy chain from the water phase into the inner part of the micelle at the temperature of 25 °C and pH ≈ 4.5–5.0, was found to be −2.39 kJ/mol. The experimentally determined *cmc* values showed good correlations with the predicted values of the bulkiness of the alkoxy tail expressed as the molar volume of substituent R, as well as with the surface tension of the compounds.

## 1. Introduction

Amphipathics are synthetic or natural molecules that covalently bond hydrophilic (soluble in water) and hydrophobic (insoluble in water) groups. In aqueous solution, such molecules self-assemble into aggregates of diverse forms and sizes [1]. These self-aggregated structures are habitually spherical assemblies [2,3,4], where the non-polar amphipathic tail groups are disposed within a hydrophilic shell provided by the polar head groups of the amphipathics, and the created species is called the micelle.

Particularly spherical micelles can increase one-dimensionally into the cylindrical, or two-dimensionally into discoidal ones, or bilayers. This process depends on a solution and its conditions, as well as on the character of a surfactant. Surfactant’s polar groups ultimately control micelle extension. It happens because both one-dimensional growths cause that the polar groups to get closer to each other. This led to restriction of the available area of a surfactant molecule at the micelle surface and influenced curvature of the surface as well [5,6].

The surfactant molecules in aqueous solutions of these micelle structures are directed with their polar groups towards the aqueous phase, and with their hydrophobic fragments outside of this phase. Regarding ionic micelles, an interphase area between aqueous and micelle phases includes ionic polar groups, the Stern layer of the electrical double layer, which was merged with these groups, approximately a half of a counter ion connected with both aqueous environment and micelle. The leftover counter ions are surrounded within the Gouy–Chapman ratio of the double layer, which goes further into the aqueous phase. In the case that the electrolytes are present, double layer length is a function of ionic power of the solution, and can be extremely compressed.

The Gibbs free increments per methylene group by different phase transitions ΔG*(CH_2_) can be provided as measuring the strength of the interactions between species in aqueous solutions of surfactant. For any homologous series of surfactants, the relationship between the critical micellar concentration *cmc* and the number of carbon atoms *n_c_* in the hydrophobic group is defined as

ln(*cmc*) = *A* − *B n_c_*, (1)

where *A* and *B* are constants reflecting the free energy changes caused in transferring the hydrophilic group and a methylene component of the hydrophobic group, respectively, from an aqueous surrounding to the micelle. The change in Gibbs free energy of transfer of any methylene group between the phases (system of two water-immiscible or partially water-miscible phases, respectively, for water (*w*) and micellar (*m*) phase at constant temperature (*T*), ΔG*(CH_2_), is given by [7,8,9]

ΔG*(CH_2_) = −*BRT*, (2)

where *R* is the universal gas constant (8.3144598 JK^−1^mol^−1^) and T is the absolute temperature.

There are various methods, like tensiometry, conductometry, fluorimetry, calorimetry, light scattering, and nuclear magnetic resonance (NMR) spectroscopy for determination of the critical micellar concentration (*cmc*) [10,11,12,13,14,15]. Spectral methods like absorption spectroscopy in ultraviolet/visible (UV–vis) region of spectrum, and fluorescence spectroscopy using other compounds as probes, are also used for the evaluation of *cmc* [16,17].

In this article, we applied the pyrene absorption method [16] for determining the *cmc* of the solution containing the series of selected morpholine derivatives of 2-alkoxyphenylcarbamic acid.

## 2. Results

These studied compounds (selected morpholine derivatives of 2-alkoxyphenylcarbamic acid) were evaluated on their local anesthetic potency [18]. It was found that their potency increases with the number (*n_c_*) of carbon atoms in the alkoxy tail, up to hexyloxy and heptyloxy chain, respectively, and then decreases. The loss/evident decrease of biological activity at bigger chain lengths is familiar as a cut-off effect [19,20,21]. The effect of these compounds depends on membrane activity. The long alkyl chain that gets through the membrane and disrupts its structure is essential for the biological influence of this kind of compounds. It has been found that these characteristics can have an impact on biological efficacy of similar kinds of compounds [22,23,24,25,26,27].

Generally, surfactants are known to be membrane perturbants, with hydrocarbon tail integrated with the lipid bilayer of membranes [28,29]. This integration results in a disturbance of the membrane. The size of the tail of the surfactants is supposed to add to the extension of membrane damage, as longer chains can integrate into the lipid bilayers. The lowered activity of compounds with a longer tail (cut-off effect) may be accompanied by their limited solubility. Because the tail lengthens, lipid solubility rises at a quicker rate than that of partition coefficient change (lipid/aqueous). These longer tails are with limited partitioning, thus, the concentration at the place of activity is not adequate to have an important influence on the membrane of the cell wall [30]. However, the decrease of activity with the tail reaching a particular length may also be explained like this: the final methyl group of a compound can be found in the place of the final groups of the hydrocarbon chains of lipids that create the opposite monolayer, where surfactants are not integrated [20]. The presence of such “sewing” of both monolayers [14], and the terminal composition, can show a raised stability and lowered ability of biological action. This feature is usually named interdigitation, and has been studied for some amphiphilic compounds, alcohols, anesthetics, and other agents [31].

Surfactants are characterized by surface properties, therefore, molar volume (MV [cm^3^]) and surface tension (ST [dyne/cm]) were predicted by means of ACD/Percepta (Advanced Chemistry Development, Inc., Toronto, Canada). This software does not predict ionic compounds, therefore, both descriptors were calculated for 2-(morpholin-4-yl)ethyl (2-alkoxyphenyl)carbamates. The values are shown in Table 1.

**Figure 1:**
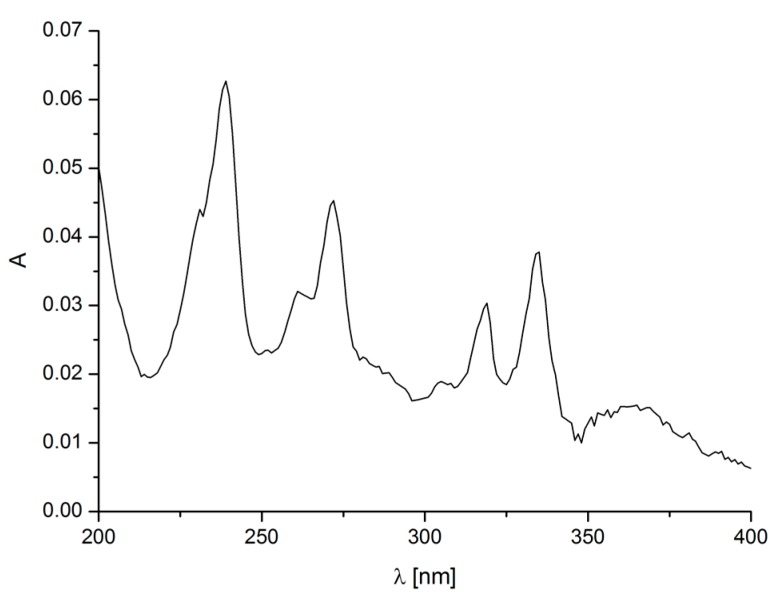
Absorption spectrum of pyrene in water (2 μM) at 25 °C.

In general, hydrophobic probes enter the micellar core and hydrophilic probes localize at the micellar surface. Thus, micellar structure may be examined by using a probe designed to localize in a particular micellar zone. This incorporation causes changes in intensities, respectively, in ratio of intensities of the peaks, in the characteristic absorption and emission spectra of the probe. The absorption spectrum of pyrene in water has showed eight peaks: strong (s) and weak (w) at 232w, 242s, 252w, 260w, 272s, 308w, 320s, and 336s nm, as depicted in Figure 1 [16,32,33]. The concentration of used pyrene was 2 µM, which was within its solubility limit of 2–3 µM. Excimer formation was expected to be absent at this concentration [16,34].

In the absorption spectra of the studied compounds in water with pyrene concentration of 2 μM, the characteristic pyrene peaks at wavelengths of 336 and 320 nm were visible. In addition, the peak at 308 nm was also visible in derivatives **6** and **7**. The other pyrene peaks were not observed due to the strong absorption of these surfactants in the near UV region, and thus, they were masked.

**Figure 2:**
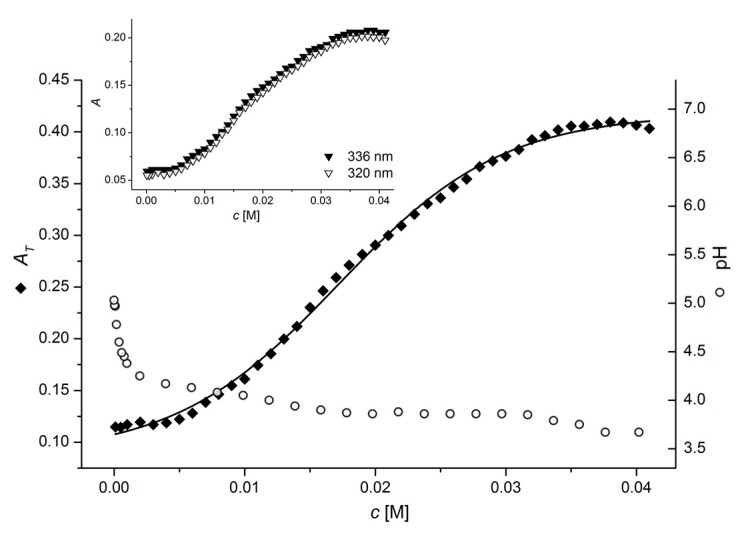
*A_T_* vs. concentration (*c*) profile for compound **4** in aqueous medium at 25 °C (◆); pH values vs. concentration (*c*) profile at 25 °C (○). Inset: Plots of absorbances (A) of individual pyrene peaks (at 336 and 320 nm) vs. surfactant concentration.

Plots of the sum of absorbances of all major pyrene peaks (*A_T_*) against the surfactant concentration (*c*) have been found to be increasingly sigmoidal in nature (see Figure 2). The absorbances of each peak were also plotted separately against surfactant concentration in the inset.

Fitting the *A_T_* vs. concentration (*c*) profiles to the sigmoidal Boltzmann equation (SBE) was herein employed for *cmc* calculation [16,17,34,35,36]. Hence,

(3) $$A_{T}=\frac{\left(a_{i}-a_{f}\right)}{1+e^{\left(x-x_{0}\right)/\Delta x}}+a_{f},$$

where *x* is the overall concentration of surfactant, *a_i_* and *a_f_* are the upper and lower limits of the sigmoid, respectively, *x*_0_ is the center of the sigmoid, and Δ*x* is directly related to the independent variable range where the abrupt change of the dependent variable occurs. The sigmoidal plot can produce two singular values of *cmc*, one at *x*_0_ and the other at (*x*_0_ + 2Δ*x*), respectively. Further, the ratio *x*_0_/Δ*x* facilitates the decision which one is the right *cmc* [16,17]. The surfactant systems that provide *x*_0_/Δ*x* < 10 produce *cmc1* = *x*_0_, and those which yield *x*_0_/Δ*x* > 10 by the SBE process produce *cmc2* = (*x*_0_ + 2Δ*x*). For aqueous solutions of all studied derivatives, the ratio *x*_0_/Δ*x* had the value of less than 10, indicating that the *cmc* is considered to be *cmc1*, and takes the value *x*_0_ according to Aguiar’s theory [17]. The parameters of each sigmoidal function (the number of fitting points *z*, the number of carbon atoms in a hydrophobic tail *n_c_*, *x*_0_, Δ*x*, the ratio *x*_0_/Δ*x*, the coefficient of determination *r^2^*, chi-square χ^2^, value *cmc1*, *cmc2*, and *cmc*) are listed in the summary Table 1.

**Figure 3:**
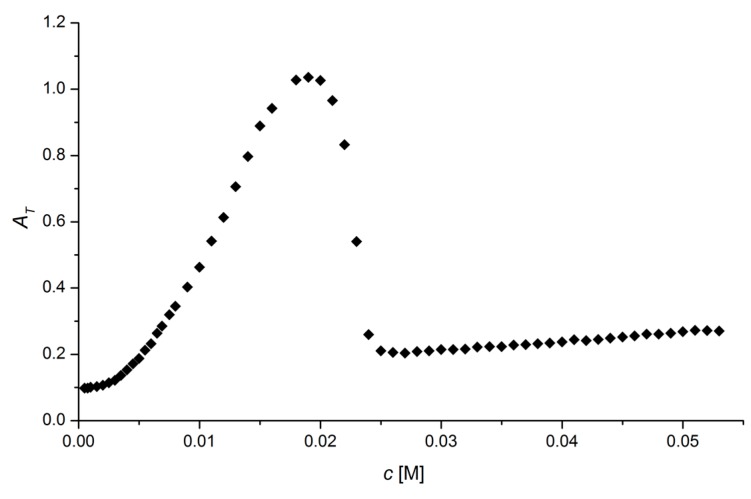
*A_T_* of main pyrene peaks at 336 and 320 nm vs. concentration (*c*) profile for compound **5** in aqueous medium at 25 °C in whole measured concentration range.

**Figure 4:**
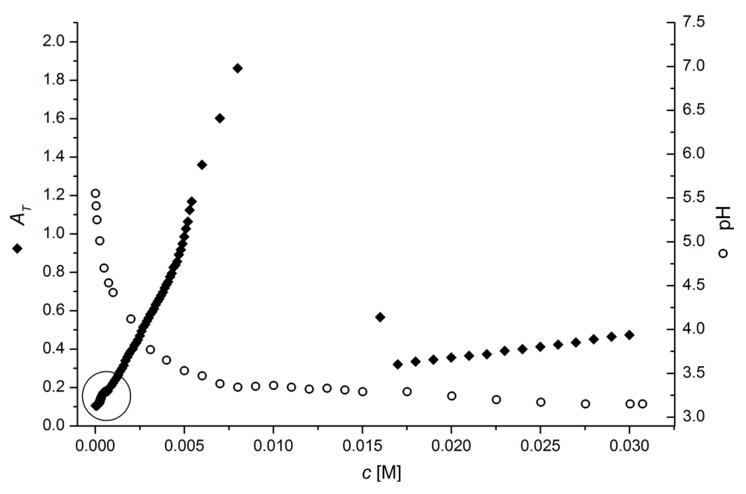
*A_T_* of main pyrene peaks at 336 and 320 nm vs. concentration (*c*) profile for compound **6** in aqueous medium at 25 °C in whole measured concentration range (◆); circle indicates concentration range where *cmc* was determined; pH values vs. concentration (*c*) profile at 2 °C (○).

**Figure 5:**
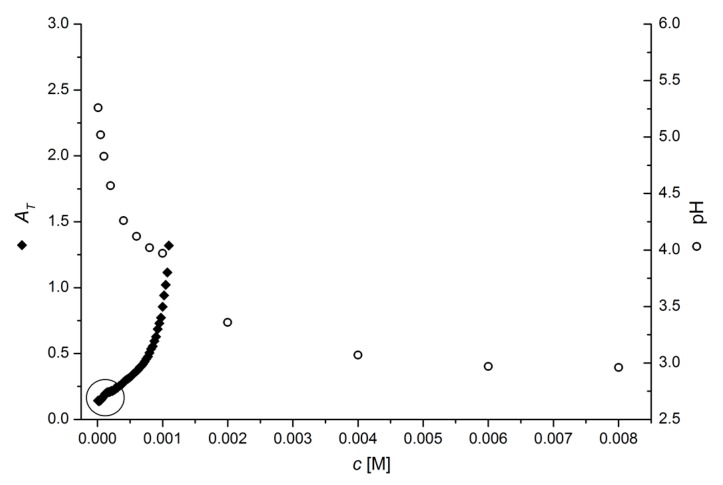
*A_T_* of main pyrene peaks at 336 and 320 nm vs. concentration (*c*) profile for compound **7** in aqueous medium at 25 °C in whole measured concentration range (◆); circle indicates concentration range where *cmc* was determined; pH values vs. concentration (*c*) profile at 25 °C (○).

During the study of substances **5**, **6**, and **7**, the significantly tarnished solutions occurred throughout the successive dilution process. At that moment, the absorbances significantly increased, and the solutions were not measurable until reaching the particular concentration at which a clear, and again, measurable solution was obtained; see Figure 3, Figure 4 and Figure 5. Only in this low concentration range was there located the inflection point which determined the *cmc* (Figure 4 and Figure 5).

One hypothesis for significant opalescence changes in solutions of compounds **5**, **6**, and **7** was the change in the pH value. Therefore, the further research has been moving in this direction. Dependence of the pH value on the concentration (*c*) of compounds **6** and **7** in aqueous solutions (Figure 4 and Figure 5) showed that the pH was unlikely to have an effect on opalescence change, because (a) the pH value was almost constant before and during the tarnishing process, and (b) the same pH vs. concentration (*c*) profile was also seen for compound **4** (see Figure 2), where the opalescence change was not seen at all.

Another hypothesis for this process was that the phase transition and the change in micelle shape from a lower to higher level of complexity occurred at a certain concentration of surfactant in the solution. This hypothesis might be the basis for further examination to confirm this apparent change, for example, using the small angle neutron scattering (SANS) that could describe the structure of molecules and aggregates in solution [37,38].

**Figure 6:**
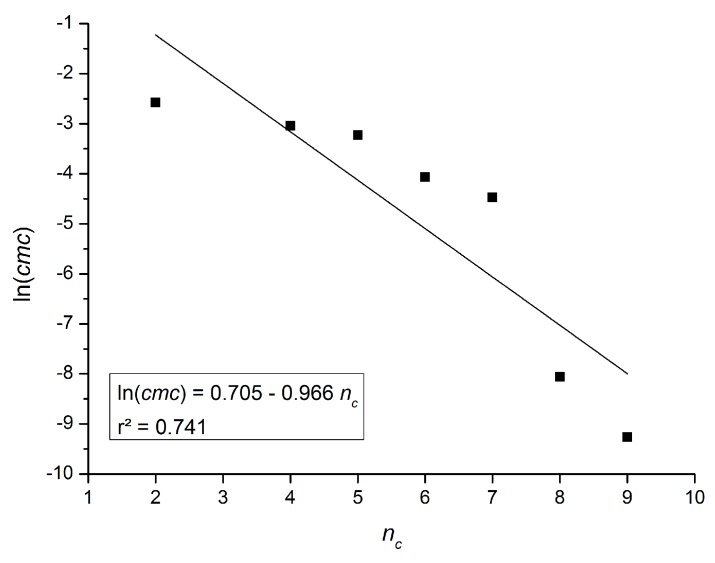
Dependence of ln(*cmc*) values on *n_c_* in hydrophobic chain.

In this study, the ln(*cmc*) values were calculated, and the plot of ln(*cmc*) vs. the number of carbon atoms, *n_c_*, in the hydrophobic chain is illustrated in Figure 6. The critical micellar concentration values decreases with increasing number of carbon atoms.

A study of micellization of the homologues of 2-alkoxyphenylcarbamoyloxyethyl-pyrrolidinium [39] and 2-alkoxyphenylcarbamoyloxyethylpiperidinium chloride [40] compared with 2-alkoxyphenylcarbamoyloxyethylmorpholinium chloride homologues in the distilled water medium and at temperature 25 °C showed that the *cmc* values increase in the order pyrrolidino- ˂ morpholino- ˂ piperidinoethyl derivatives. The most probable reason is the presence of the oxygen atom in the morpholine cycle of side chain, which remarkably changes the lipophilicity of the entire molecule in favor of its hydrophilic properties, which lead to increased solubility in water. Increased solubility in water can create a better environment for the formation of quantitative and qualitative parameters of micelles.

The ln(*cmc*) values of compounds with the number of carbon atoms *n_c_* = 2, 4, 5, 6, 7, 8, and 9 are linearly dependent on *n_c_*: ln(*cmc*) = 0.705 − 0.966 *n_c_* with the coefficient of determination *r*^2^ = 0.741. The *cmc* values decrease with increasing number of carbon atoms. However, it seems that the ln(*cmc*) dependence on *n_c_* deviates from linearity for substances *n_c_* = 8 and 9, consequently, there is a low value for the coefficient of determination (*r*^2^ = 0.741). In this case, the curve could probably have a quasi-parabolic dependence. This deviation is presumably due to longer alkoxy chain and the decreased solubility of these substances. They form micelles at low concentrations ranging from 10^−5^–10^−4^ M, as compared to homologues having a lower number of carbon atoms in the alkoxy chain, which form micelles at concentrations over 10^−2^ M. A large difference between these concentration values could probably cause the deviation from the linearity. The obtained experimental results support a model, according to which the quasi-parabolic dependencies of biological potencies of amphiphilic compounds on the length of their hydrophobic substituent (“cut-off” effect) could be caused by structural defects in lipid bilayer of the membrane induced by amphiphilic compounds or by micellization [19,40]. The Gibbs free energy ΔG*(CH_2_), necessary for the transfer of the methylene group of the hydrophobic chain from an aqueous phase into the micelle interior at the temperature of 25 °C and pH ≈ 4.5–5.0, has, according to Equation (2), the value of −2.39 kJ/mol. For alkyloxy homologues of local anesthetic heptacainium chloride determined by the ion-selective electrode, the value of ΔG*(CH_2_) in water and in the 0.1 M NaCl solution is −1.21 kJ/mol and −1.54 kJ/mol, respectively [41]. It is assumed that the differences between these two results might be caused by the different species of the polar groups of two anesthetics, and by the composition of the system.

Using the Equations (1) and (2), and the experimental value of B = 0.966 given in Figure 6, the free energy change ΔG*(CH_2_), involved in the transfer of the methylene group of the hydrophobic chain from an aqueous phase into the micelle internal, is negative, accordingly favoring micellization. This accounts also for the fact that the *cmc* values decrease with increasing length of the hydrophobic group. Using Equation (1) and the value of A = 0.705 depicted in Figure 6, the free energy change, involved in the transport of the hydrophilic group from an aqueous surroundings to the micelle external, is positive, and then goes up against micellization.

**Figure 7:**
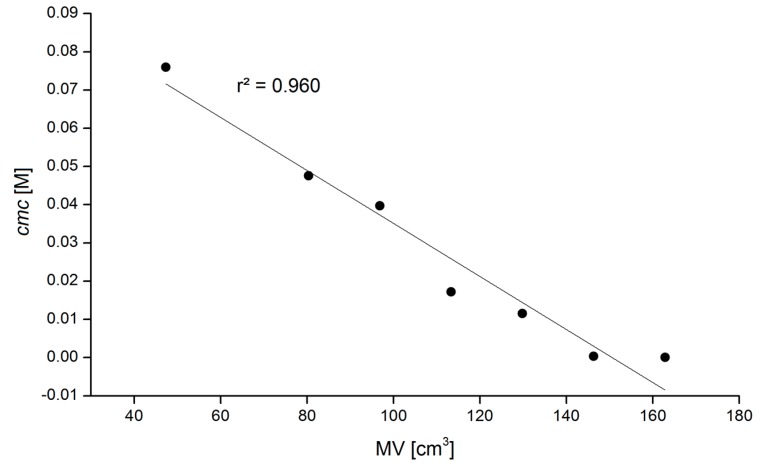
Dependence of *cmc* values on bulkiness alkoxy tail expressed as molar volume (MV [cm^3^]) of substituent R.

**Figure 8:**
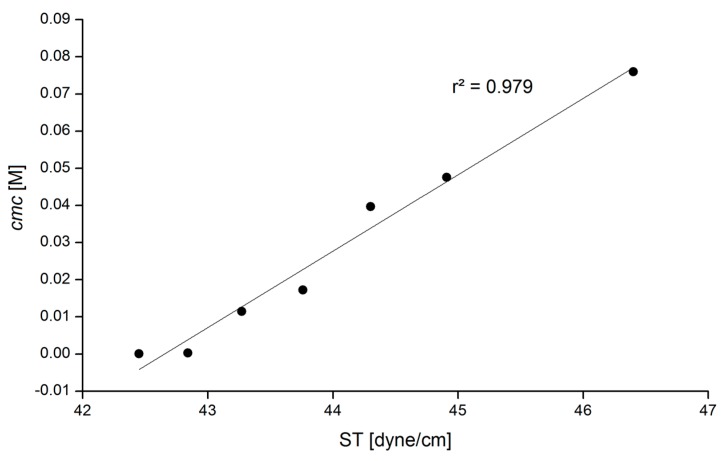
Dependence of *cmc* values on surface tension (ST [dyne/cm]) of whole compound.

As mentioned above, the discussed compounds can be considered as surfactants, i.e., their biological effect is connected with the increasing surface activity (i.e., a longer tail as R substituent is preferable). This parameter is reflected in the bulkiness/molar volume (MV [cm^3^]) of the alkoxy chain (substituent R). The surface tension (ST [dyne/cm]) of the compound is also connected with the length of the alkoxy tail. The calculated values of surface tension increase (i.e., surface activity decreases) with decreasing bulkiness, expressed as molar volume (reflecting a decrease in the length of the alkoxy tail). The dependence of the *cmc* values of the discussed compounds on the bulkiness/molar volume (MV [cm^3^]) of the alkoxy chain is illustrated in Figure 7. It can be stated that the *cmc* values of the compounds linearly decrease (*n_c_* = 7, r^2^ = 0.960) with increasing bulkiness (the length of the tail). On the other hand, but logically, the *cmc* values linearly increase (*n_c_* = 7, r^2^ = 0.979) with increasing surface tension (i.e., with a decrease of surface activity), see Figure 8, where the dependence of the *cmc* values on the surface tension of the compounds is illustrated. These linear dependences showed in Figure 7 and Figure 8 underline the accuracy of the determined *cmc* values of the discussed compounds and their usefulness for investigation of compounds of a similar type. Our results showed a close relationship between the experimentally determined *cmc* and surface activity, respectively, biological activity of the compounds.

## 3. Materials and Methods

### 3.1. Chemistry

**Scheme 1:**
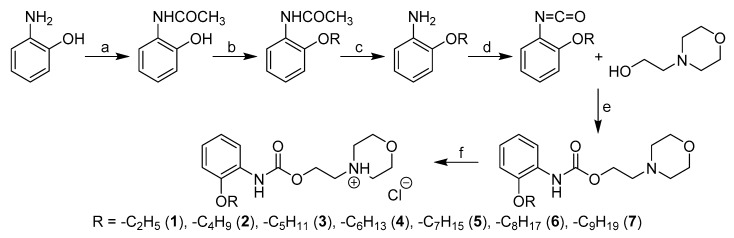
Synthesis of 4-(2-{[(2-alkoxyphenyl)carbamoyl]oxy}ethyl)morpholin-4-ium chloride **1**–**7**. *Reagents and conditions*: (**a**) (CH_3_CO)_2_O, room temperature, 1 h; (**b**) Na, EtOH, alkylbromides, reflux, 3 h; (**c**) 18% HCl, reflux, 2 h; (**d**) COCl_2_, toluene, reflux, 3 h; (**e**) toluene, reflux, 3 h; (**f**) HCl, Et_2_O [10,11].

The investigated compounds were prepared according to Scheme 1 by Cizmarik et al. [18,42]. Briefly, the starting material 2-aminophenol with acetanhydride yielded 2-acetaminophenol that, by condensation with appropriate alkylbromide, gave a series of seven alkoxyacetanilides, the cleavage of which by an aqueous solution of hydrochloric acid provided the corresponding alkoxyanilines. By reaction with phosgene in anhydrous toluene, the latter yielded alkoxyphenylisocyanates, that gave 2-(morpholin-4-yl)ethyl (2-alkoxyphenyl)carbamates by reaction with 2-morpholinoethan-1-ol. Hydrochloride salts **1–7** (see Scheme 1 and Table 1) of these carbamates were prepared using an equimolar amount of ethereal hydrogen chloride.

Finally, studied compounds **1**–**7** were recrystallized before investigations. All studied compounds are white crystalline substances. The structural identity and purity of each of compounds were verified using high-resolution mass spectrometry, and were measured using a high-performance liquid chromatograph Dionex UltiMate^®^ 3000 (Thermo Scientific, Waltham, MA, USA) coupled with a LTQ Orbitrap XL^TM^Hybrid Ion Trap-Orbitrap Fourier Transform Mass Spectrometer (Thermo Scientific, Waltham, MA, USA) with injection into HESI II in the positive or negative mode. Pyrene (≥99%, Sigma-Aldrich, Buchs, Switzerland). Experimental assays were performed at laboratory temperature 25 °C. The critical micellar concentration (*cmc*) values were established by methods [16,17].

### 3.2. Preparation of Pyrene Stock Solution

All investigated compounds **1**–**7** were easily soluble in the water. A store solution of pyrene was prepared by adding a known weight of the compound in 20 wt % ethanol in water. The mixture was sonicated in order to prepare a clear solution. Its dilution provided the experimental 2 µM solution of pyrene, in which a concentration of ethanol was 0.25%. Such a minimal concentration of ethanol was considered unable to influence the self-aggregation processes and spectral performance of amphiphiles as well.

### 3.3. Absorbance Measurements

An absorbance study was performed by UV–vis spectrophotometer Spekol 1300 (Analytic Jena AG, Jena, Germany) using 10 mm path length quartz cuvette. The spectra were recorded in the 200–400 nm wavelength scope.

### 3.4. pH Measurements

pH values of studied local anesthetics were measured by inoLab Cond 7110 pH meter (Xylem Analytics Germany Sales GmbH & Co., KG, WTW, Weilheim, Germany).

## 4. Conclusions

Critical micellar concentrations within a homologous series of 4-(2-{[(2-alkoxyphenyl)carbamoyl]oxy}ethyl)morpholin-4-ium chlorides were determined. These compounds were prepared as local anesthetics, i.e., surfactant-type compounds; therefore, their surface activity characterization is important for their biological potential. UV absorption of pyrene in surfactant solution has been an easy method to establish the *cmc* of surfactant. The values of *cmc* with the number of carbon atoms *n_c_* = 2, 4, 5, 6, 7, 8 ,and 9 linearly dependent on *n_c_*: ln(*cmc*) = 0.705 − 0.966 *n_c_*. The Gibbs free energy change ΔG*(CH_2_), necessary for the transfer of the methylene group of the hydrophobic chain from an aqueous phase into the micelle interior, has a value of −2.39 kJ/mol at 25 °C. It can be provided as a measure of the intensity of the interactions between species in aqueous solutions of surfactant. The experimentally determined *cmc* values showed good correlations with the predicted values of the bulkiness of the alkoxy tail, expressed as the molar volume (MV [cm^3^]) of substituent R, as well as with the surface tension (ST [dyne/cm]) of the compounds.

## Figures and Tables

**Table 1:** Structures of investigated 4-(2-{[(2-alkoxyphenyl)carbamoyl]oxy}ethyl)morpholin-4-ium chloride **1**–**7**, parameters of fitting functions with determined critical micellar concentration (*cmc*) values at 25 °C, and predicted values of molar volume (MV [cm^3^]) of substituent R and surface tension (ST [dyne/cm]).

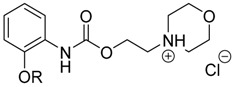													
Comp.	R	*n_c_*	*z*	*x*_0_	Δ*x*	*x*_0_/Δ*x*	r^2^	χ^2^	*cmc1* [M]	*cmc2* [M]	*cmc* [M]	MV * [cm^3^]	ST * [dyne/cm]
**1**	C_2_H_5_	2	28	0.0760	2.17 × 10^−2^	3.50	0.998	8.19 × 10^−6^	7.60 × 10^−2^	1.19 × 10^−1^	7.60 × 10^−2^	47.29	46.40
**2**	C_4_H_9_	4	42	0.0476	7.90 × 10^−3^	6.03	0.995	2.89 × 10^−6^	4.76 × 10^−2^	6.34 × 10^−2^	4.76 × 10^−2^	80.31	44.91
**3**	C_5_H_11_	5	69	0.0397	2.76 × 10^−2^	1.44	0.998	3.33 × 10^−5^	3.97 × 10^−2^	9.49 × 10^−2^	3.97 × 10^−2^	96.81	44.30
**4**	C_6_H_13_	6	42	0.0172	6.29 × 10^−3^	2.73	0.998	2.63 × 10^−5^	1.72 × 10^−2^	2.98 × 10^−2^	1.72 × 10^−2^	113.32	43.76
**5**	C_7_H_15_	7	28	0.0115	3.26 × 10^−3^	3.52	0.998	2.26 × 10^−4^	1.15 × 10^−2^	1.80 × 10^−2^	1.15 × 10^−2^	129.83	43.27
**6**	C_8_H_17_	8	29	3.17 × 10^−4^	7.10 × 10^−5^	4.46	0.999	2.59 × 10^−6^	3.17 × 10^−4^	4.59 × 10^−4^	3.17 × 10^−4^	146.33	42.84
**7**	C_9_H_19_	9	21	9.47 × 10^−5^	2.80 × 10^−5^	3.38	0.995	1.40 × 10^−5^	9.47 × 10^−5^	1.51 × 10^−4^	9.47 × 10^−5^	162.84	42.45

* Predicted using of ACD/Percepta v. 2012 (Advanced Chemistry Development, Inc., Toronto, ON, Canada, 2012) not for final hydrochlorides but for 2-(morpholin-4-yl)ethyl (2-alkoxyphenyl)carbamates.
